# Supplementary figures and images for: The association of telomere length and telomerase activity with adverse outcomes in older patients with non-ST-elevation acute coronary syndrome
Source: PLoS One. 2020 Jan 10;15(1):e0227616. doi: 10.1371/journal.pone.0227616 (PMC6953865; doi:10.1371/journal.pone.0227616)

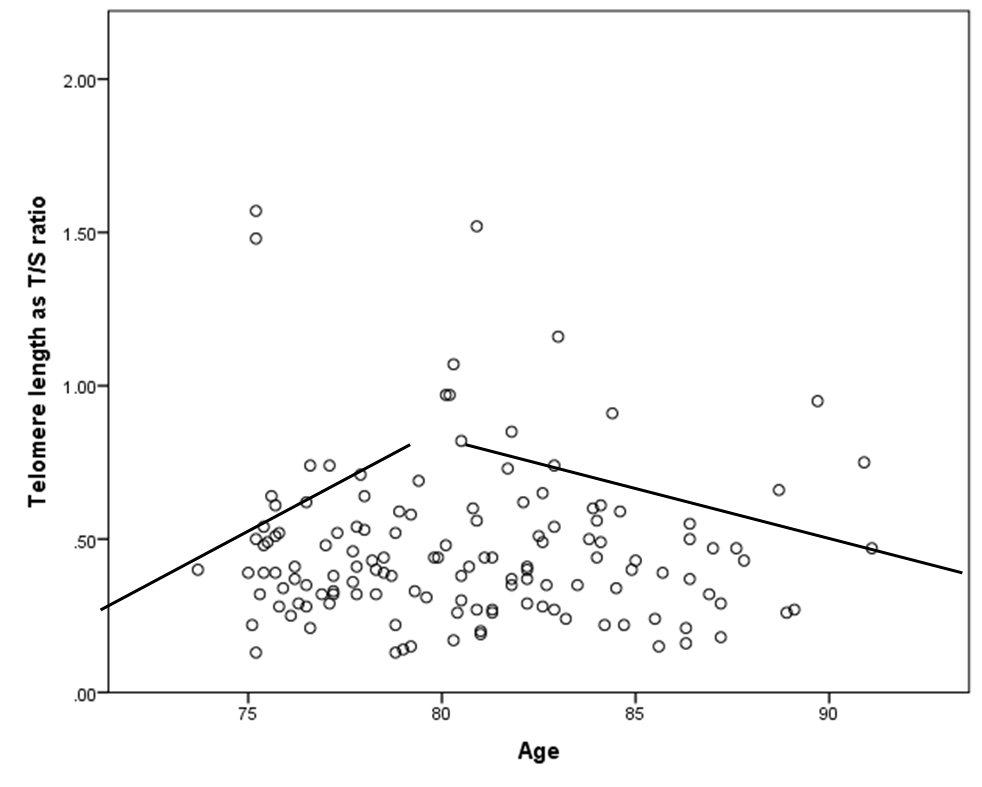

Supplement: S1 Fig — Scatter plot analysis of TL with age yields a non statstiacally significant increasing regression line in aged 75–80 (Spearman's correlation coefficient, rs = 0.052, p = 0.66) and a decreasing regression line in aged > 80 (rs = -0.11, p = 0.34). (TIF) [file pone.0227616.s001.tif]

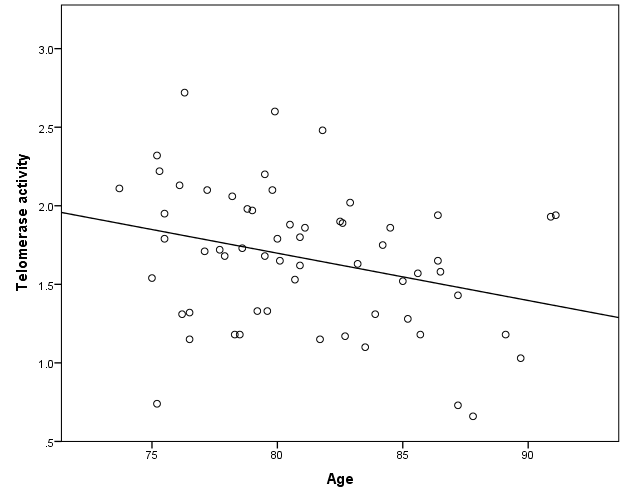

Supplement: S2 Fig — Scatter plot analysis of TA with age yields a non-statistically significant decreasing regression line (Spearman's correlation coefficient, rs = -0.22, p = 0.071). (TIF) [file pone.0227616.s002.tif]

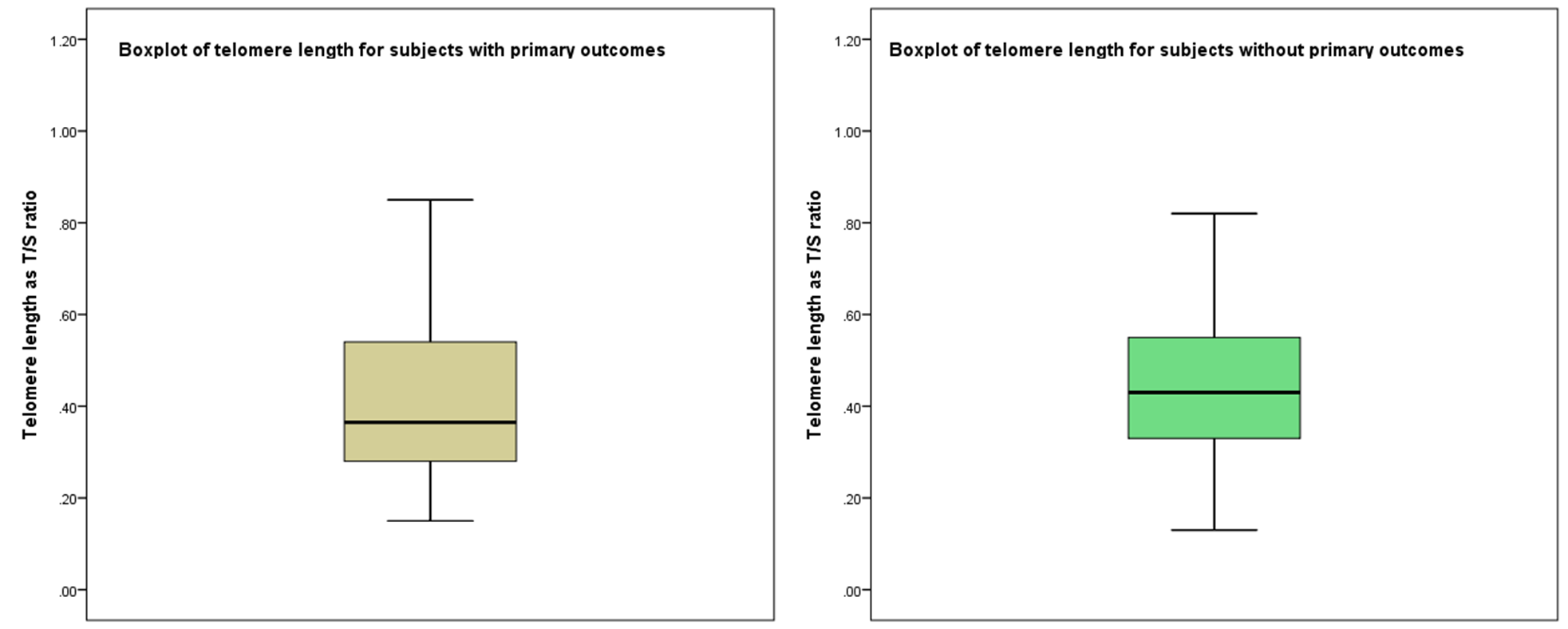

Supplement: S3 Fig — A Wilcoxon signed–rank test showed that telomere lengths did not elicit a significant change for subjects with primary outcomes compared to subjects without composite events (Z = -0.655, p = 0.071). (TIF) [file pone.0227616.s003.tif]

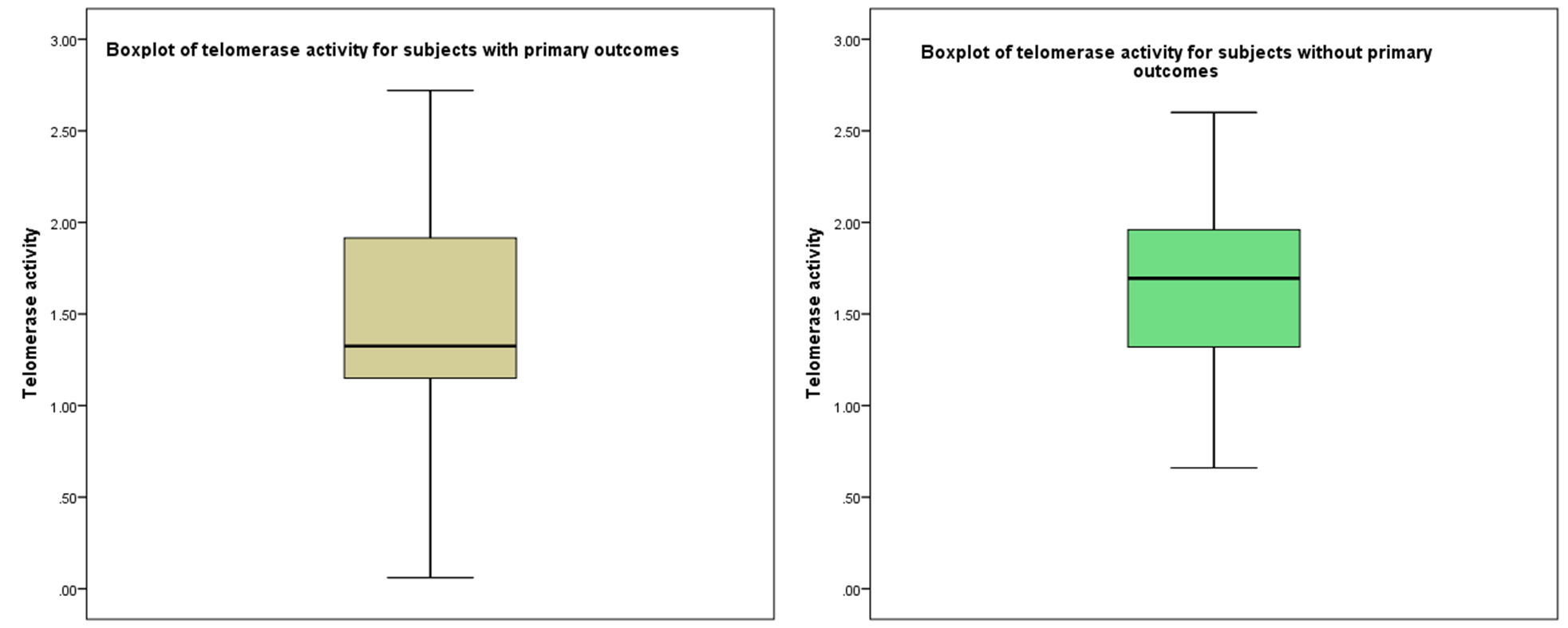

Supplement: S4 Fig — A Wilcoxon signed–rank test showed that telomerase activity did not elicit a significant change for subjects with primary outcomes compared to subjects without composite events (Z = -1.274, p = 0.20). (TIF) [file pone.0227616.s004.tif]
