# Supplementary material for: The association of telomere length and telomerase activity with adverse outcomes in older patients with non-ST-elevation acute coronary syndrome
Source: PLoS One. 2020 Jan 10;15(1):e0227616. doi: 10.1371/journal.pone.0227616 (PMC6953865; doi:10.1371/journal.pone.0227616)
Supplement: S1 Table — Cox regression analysis preformed for combined and primary outcomes alone using telomere length and telomerase activity as predictors. Both predictors were divided into two groups for analysis based upon area under curve (ROC). For TL, area under curve was 0.57 (p = 0.17). The best cutoff was 0.61 with sensitivity of 87% and specificity of 80%. With this cut-off, 111 participants had STL (82.2%) and 24 had LTL (17.8%). The area under curve was also measured for TA at 0.54 (p = 0.57), with cut-off of 1.88 (sensitivity of 80% and specificity of 64%). 47 (70.1%) participants classified as low TA and 20 (29.9) as high TA. * LTL used as reference. † High used as reference. TL- telomere length and TA-telomerase activity. (DOCX) [file pone.0227616.s005.docx]

|  | **Hazard ratio** | **95% confidence interval** | ***p*-value** |
| --- | --- | --- | --- |
| TL* | 1.42 | 0.56 – 3.65 | 0.46 |
| TA† | 1.87 | 0.70 – 4.99 | 0.21 |
