# Supplementary material for: The association of telomere length and telomerase activity with adverse outcomes in older patients with non-ST-elevation acute coronary syndrome
Source: PLoS One. 2020 Jan 10;15(1):e0227616. doi: 10.1371/journal.pone.0227616 (PMC6953865; doi:10.1371/journal.pone.0227616)
Supplement: S2 Table — Cox regression analysis preformed for combined outcomes without major bleeding using telomere length and telomerase activity as predictors. Both predictors were divided into tertiles for analysis. * LTL used as reference. † High used as reference. MTL- medium telomere length, STL-short telomere length and TA-telomerase activity. (DOCX) [file pone.0227616.s006.docx]

|  | **Hazard ratio** | **95% confidence interval** | ***p*-value** |
| --- | --- | --- | --- |
| TL* |  |  |  |
| MTL | 0.46 | 0.16 – 1.36 | 0.46 |
| STL | 0.92 | 0.38 – 2.22 | 0.86 |
| TA† |  |  |  |
| Mid | 1.04 | 0.15 – 7.39 | 0.97 |
| Low | 3.40 | 0.68 – 16.86 | 0.13 |
