# Supplementary material for: The association of telomere length and telomerase activity with adverse outcomes in older patients with non-ST-elevation acute coronary syndrome
Source: PLoS One. 2020 Jan 10;15(1):e0227616. doi: 10.1371/journal.pone.0227616 (PMC6953865; doi:10.1371/journal.pone.0227616)
Supplement: Attachment — Submitted filename: Response to reviewers.docx [file pone.0227616.s007.docx]

Reviewer #1: 1. Introduction: Previous data is well summarized and the main aim of the study is clearly specified.

2. Methods:

-Patients were selected from ICON1 study. Authors affirmed that patients aged > 65 years old were included. Maybe this cut-off point does not reflect the real “old patients”, in addition mean age of the patients included in this study is around 80 years old. Did you selected very old patients from the whole sample of ICON1 study or you included any patients older than 65 years old?

*We agree that older patients would ideally have a greater age cut off. However, we believe that the mean age of participants in the study of 80 years does reflect older patients within this population. We did not specially select very old patients in this study, but included all patients older than 65 with available data for analysis from ICON-1 study.*

-You divided the sample in tertiles. This is completely correct but it might be more precise to do it according to the best cut off point calculated by ROC curve. In this way you can provide data of sensitivity and specificity.

*We have performed the analysis by dividing the predictors based upon area under curve (ROC). For TL, area under curve was 0.57 (p = 0.17). The best cutoff was 0.61 with sensitivity of 87% and specificity of 80%. With this cut-off, 111 participants had STL (82.2%) and 24 had LTL (17.8%). The area under curve was also measured for TA at 0.54 (p =0.57), with cut-off of 1.88 (sensitivity of 80% and specificity of 64%). 47 (70.1%) participants classified as low TA and 20 (29.9) as high TA. Telomere and telomerase activity were not predictive of primary outcomes. The results can be found in Table S5.*

-You designed a composite endpoint with thrombotic and bleeding events. In previous studies, telomere length seems to be connected to atherosclerosis and the evidence about its relationship with bleeding is weaker. It could be interesting to calculate a secondary combined endpoint formed only by thrombotic events.

*We have performed the analysis using thrombotic events only as a secondary combined outcomes. The results suggest that Telomere and telomerase activity were not associated with the secondary outcomes. Details of this result can be seen in table S6.*

3. Results

-The incidence of adverse events is too low in your patients. Previous studies of very old patients with ACS showed 6 months mortality around 12% (Alegre et al. J Am Med Dir Assoc. 2017 Nov 17).

*We agree that the incidence of adverse events in our study is relatively low, which is highlighted in the study limitations.*

-Please, comment the percentage of AMI and unstable angina in your sample.

*Of the study participants, 109 (82%) with admitted with AMI, and 26 (18%) presented with unstable angina. Index presentation has been added to table 1.*

-Could you provide the incidence of mortality caused by cardiovascular causes in your sample?

*Unfortunately, we did not have the detail of the specific cause of mortality in this study population.*

-Do you have data about the characteristics of the coronary event of your patients (number of affected arteries, Killip class, number of stents used…)? Is the peak value the troponin measurement provided en table 1?

*Procedural data has now been added and can be found in table 2. Peak troponin measurements can be found in table 1. Telomere length was not associated with the characteristics of the coronary event in the study population.*

4. Discussion

--In the reference 25 of your manuscript (Epel et al.), telomere attrition but not initial telomere length was associated to cardiovascular mortality. In your study you did not find any relationship between telomerase activity and mortality. Could you explain in detail how your results agree or disagree with this paper?

*Epel and colleagues found that the rate of telomere shortening was predictive of mortality from cardiovascular disease in elderly men, suggesting that low telomerase activity contribute to cardiovascular risk. However, our study found that telomerase activity was not associated with adverse outcomes following invasive management of NSTEACS. This may partly be explained by the difference of study populations. Epel et al. recruited healthy older volunteers prior to cardiovascular event, compared to the patients presenting with NSTEACS in the current study. Hence, this study focused on telomere length in those whom already developed coronary artery disease. The results together would suggest that dependent upon the cumulative oxidative stress and inflammatory burden, telomere attrition may play a different role in the process of atherosclerosis in older patients.*

-Other papers have studied the prognosis effect of telomere length in older patients with ACS (Am J Cardiol 2014;113:418e421). In this paper telomere length showed a prognostic effect in middle aged men with ACS but not in older patients. The group of elderly patients in this study was small (n: 52), presented STEMI and NSTEMI and revascularization was performed only in the 53% of patients so the sample is quite different to yours. Nevertheless, results are similar so it might be interesting to discuss this paper in your manuscript.

*Perez-Rivera et al. found worse prognosis with short telomere length in middle aged men with ACS but not in older patients, which is consistent with the result of the current study. This would add further evidence that telomere attrition may play a different role in the process of atherosclerosis in older patients. This have been added to the discussion section of the manuscript.*

Reviewer #2: The Authors presented a substudy of the ICON-1 Biomarker study, aiming to determine the possible Association between telomere length and telomerase activity with the outcome of old patients presenting with NSTEACS.

The manuscript is well written and addresses a clinically relevant issue with potential translational relevance.

However, there are major issues to be resolved before considering the paper subitable for pubblication.

- was this substudy pre-specified in the trial design? If not, this could be a relevant methodological bias.

*This substudy was planned in the ICON-1 study, and has now been included in the methods section.*

- are the patients recruited consecutive?

*In the ICON1 study, consecutive sampling was used. Participants were recruited from patients referred to two tertiary cardiac care hospitals from the neighbouring district general hospitals for invasive treatment of NSTEACS.*

- the reason why almost half of the patients recruited in the general study have been esclude in this substudy is clear and not reported. Please add. It is also relevant to explain Why Only in 67 patients telomerase activity data were available. These may be considered profound bias in the analysis of the data presented.

*Only 135 participants had appropriate samples for testing telomere length and due to lack of funding, only 67 of all available samples could be analysed for telomerase activity.*

- The Authors stated that these patients underwent an invasive management of the NSTEACS. How many of these underwent PCI? Procedural data are not reported. Therapy on admission and at discharge was not reported. All these informations might have a profound impact on the data and outcome analysis.

*A total of 111 (82.2%) patients undergone PCI, with 85 (63%) patients having single vessel disease, and 27 (20%) with multi vessels disease. Further details of the procedure and medical therapy on discharge has now been added and can be found on table 2. No significant association was observed between telomere length and procedure or medical therapy.*

- the division in to tertiles seems to be arbitrary.

*Telomere lengths was divided into tertile as it allows for the analysis between shorter and longer telomere length, which is consistent with previous literature.*

- When considering frail vs pre-frail patients, are there any difference in the distribuition of TL and TA and their association with the outcome?

*As shown in table 1, there were no significant differences in telomere length between frail vs pre-frail patients. This is also observed in telomerase activity. Frailty was associated with outcome, as shown in the ICON1 study.*

- Recently Werner et al demonstarted that specific modalities of exercise might influence regulators of cellular senescence (i.e TL e TA). Please comment.

*Werner and colleagues found that specific exercise modalities were associated with increased telomere length and telomerase activity. This raises the interesting possibility that the benefits of exercise in reducing and preventing frailty in the older population may act through regulating cellular senescence. The current study found no association between telomere length and frailty, which is consistent with the results demonstrated by Brault et al. in older adults with cardiac disease. These results suggest that frailty may be the consequence of repeated physiological stress induced premature cellular senescence in addition to progressive telomere shortening. It would be interesting to investigate the effect of exercise on telomere length and frailty in older adults with established cardiac disease. This discussion has been added into the manuscript under the discussion section.*
